# United States Veterinary Medical Association

**Published:** 1892-09

**Authors:** R. S. Huidekoper, W. Horace Hoskins

**Affiliations:** President; Secretary; 12 South 37th Street, Phila.


### EDITORIAL.

### UNITED STATES VETERINARY MEDICAL ASSOCIATION.

PHILADELPHIA, Pa., August 22, 1892.

To all members of the United States Veterinary Medical Association and the Veterinary profession throughout the United States:

All applications for membership filed in the office with the Secretary on or before September 15th, will receive consideration at Boston on September 20th.

The Secretary desires to give notice that at least ten papers, aside from the reports of Committees, will be for consideration at this meeting, embracing subjects of the greatest interest and importance to the entire profession.

All railroads east of Chicago, and the Chicago and Alton from Bloomington, Ill., will grant the one and one-third rates to all members attending this meeting.

The meeting will be held in Union Hall, 48 Boylston Street, Boston, Mass., on September 20th, 21st, and 22d. The Committee of Arrangements recommend the following hotels: Young's Hotel, United States Hotel, Hotel Reynolds, and the Thorndike.

A special meeting of the International Committee of Arrangements will be held at Young's Hotel, September 19th, 1892, at 4 P. M. sharp. A special meeting of the Comitia Minora will be held at Young's Hotel, at 8 P. M., September 19th, 1892.

The banquet will be given at Young's Hotel; it will close our exercises at 7 P. M., on the 22d. A general invitation is extended to the entire veterinary profession of the United States to be present at this meeting.

R. S. HUIDEKOPER,
*President.*

W. HORACE HOSKINS, *Secretary.*
12 South 37th Street, Phila.

